# Real-world comparative outcomes and toxicities after definitive radiotherapy using proton beam therapy versus intensity-modulated radiation therapy for prostate cancer: a retrospective, single-institutional analysis

**DOI:** 10.1093/jrr/rrae065

**Published:** 2025-01-15

**Authors:** Yojiro Ishikawa, Motohisa Suzuki, Hisashi Yamaguchi, Ichiro Seto, Masanori Machida, Yoshiaki Takagawa, Yusuke Azami, Yuntao Dai, Nor Shazrina Sulaiman, Satoshi Teramura, Yuki Narita, Takahiro Kato, Yasuyuki Kikuchi, Yasuo Fukaya, Masao Murakami

**Affiliations:** Department of Radiation Oncology, Southern Tohoku Proton Therapy Center 7-172, Yatsuyamada, Koriyama, Fukushima 963-8052, Japan; Division of Radiology, Faculty of Medicine, Tohoku Medical and Pharmaceutical University, 1-15-1 Fukumuro, Miyagino-ku, Sendai, Miyagi 983-8536, Japan; Department of Radiation Oncology, Southern Tohoku Proton Therapy Center 7-172, Yatsuyamada, Koriyama, Fukushima 963-8052, Japan; Department of Radiation Oncology, Southern Tohoku Proton Therapy Center 7-172, Yatsuyamada, Koriyama, Fukushima 963-8052, Japan; Department of Minimally Invasive Surgical and Medical Oncology, Fukushima Medical University, 1 Hikarigaoka, Fukushima 960-1295, Japan; Department of Radiation Oncology, Southern Tohoku Proton Therapy Center 7-172, Yatsuyamada, Koriyama, Fukushima 963-8052, Japan; Department of Radiation Oncology, Southern Tohoku Proton Therapy Center 7-172, Yatsuyamada, Koriyama, Fukushima 963-8052, Japan; Department of Radiation Oncology, Southern Tohoku Proton Therapy Center 7-172, Yatsuyamada, Koriyama, Fukushima 963-8052, Japan; Department of Minimally Invasive Surgical and Medical Oncology, Fukushima Medical University, 1 Hikarigaoka, Fukushima 960-1295, Japan; Department of Radiation Oncology, Southern Tohoku Proton Therapy Center 7-172, Yatsuyamada, Koriyama, Fukushima 963-8052, Japan; Department of Radiation Oncology, Southern Tohoku Proton Therapy Center 7-172, Yatsuyamada, Koriyama, Fukushima 963-8052, Japan; Department of Radiation Oncology, Southern Tohoku Proton Therapy Center 7-172, Yatsuyamada, Koriyama, Fukushima 963-8052, Japan; Department of Radiation Oncology, Southern Tohoku Proton Therapy Center 7-172, Yatsuyamada, Koriyama, Fukushima 963-8052, Japan; Division of Radiology, Faculty of Medicine, Tohoku Medical and Pharmaceutical University, 1-15-1 Fukumuro, Miyagino-ku, Sendai, Miyagi 983-8536, Japan; Department of Minimally Invasive Surgical and Medical Oncology, Fukushima Medical University, 1 Hikarigaoka, Fukushima 960-1295, Japan; Department of Radiation Oncology, Southern Tohoku Proton Therapy Center 7-172, Yatsuyamada, Koriyama, Fukushima 963-8052, Japan; Department of Radiological Sciences, School of Health Sciences, Fukushima Medical University, 1 Hikariga-oka, Fukushima 960-1295, Japan; Department of Radiation Oncology, Southern Tohoku Proton Therapy Center 7-172, Yatsuyamada, Koriyama, Fukushima 963-8052, Japan; Department of Urology, Southern Tohoku Hospital, 7-172 Yatsuyamada, Koriyama, Fukushima 963-8052, Japan; Department of Radiation Oncology, Southern Tohoku Proton Therapy Center 7-172, Yatsuyamada, Koriyama, Fukushima 963-8052, Japan

**Keywords:** prostate cancer, intensity-modulated radiation therapy, proton beam therapy, biochemical recurrence, biochemical relapse-free survival

## Abstract

This retrospective study aimed to compare the clinical outcomes of intensity-modulated radiation therapy (IMRT) and proton beam therapy (PBT). A total of 606 patients diagnosed with prostate cancer between January 2008 and December 2018 were included. Of these patients, 510 received PBT up to a dose of 70–78 Gy (relative biological effectiveness) and 96 patients received IMRT up to a dose of 70–78 Gy. The median follow-up period was 82 months (range: 32–140 months). Patients in the PBT group had significantly higher 7-year rates of biochemical relapse-free survival (bRFS) and disease-free survival (DFS) rates: 95.1% for PBT vs 89.9% for IMRT (*P* = 0.0271) and 93.1% for PBT vs 85.0% for IMRT (*P* = 0.0019). After matching analysis, 94 patients were assigned to both groups, and the PBT group showed significantly higher 7-year bRFS and DFS rates: 98.9% for PBT vs 89.7% for IMRT (*P* = 0.023) and 93.4% for PBT vs 84.6% for IMRT (*P* = 0.022), respectively. In the subgroup analysis of intermediate-risk patients, the PBT group showed a significantly higher 7-year bRFS rate (98.3% for PBT vs 90.5% for IMRT; *P* = 0.007). The V60 of the bladder in the PBT group (18.1% ± 10.1%) was higher than that in the IMRT group (14.4% ± 7.6%) (*P* = 0.024). This study found that the treatment outcomes of PBT potentially surpassed those of IMRT specifically concerning bRFS and DFS in real-world settings. However, it should be noted that attention is warranted for late bladder complication of PBT.

## INTRODUCTION

According to the latest statistics, prostate cancer is the fourth most frequently diagnosed cancer among men in Japan, following lung, stomach and colorectal cancers. This emphasizes the need to address the high incidence of these types of cancers in Japanese men [1], and while advancements have expanded the treatment options, it remains a complex and multifaceted challenge. Among the available therapeutic approaches, proton beam therapy (PBT) and intensity-modulated radiation therapy (IMRT) have gained widespread recognition for their effectiveness and tolerability in prostate cancer management [2, 3]. However, despite favorable outcomes shown in previous studies [4, 5], a definitive consensus regarding the superiority of PBT over IMRT or vice versa remains elusive, casting a shadow over clinical decision-making and research protocol design [5]. The absence of a clear victor in this therapeutic dilemma hinders the development of clinical research protocols and complicates the choices made by physicians and patients. Furthermore, conducting randomized studies to resolve this issue is challenging, including cost-effectiveness and ethical considerations [6]. Patients’ financial capabilities and preferences often sway the choice toward PBT, particularly in regions uncovered by public insurance [7]. Additionally, previous study endeavors struggled to make accurate comparisons between the two treatment strategies due to the inherent variations in institutions and protocols employed in PBT and IMRT [4]. In response to this uncertainty and variation backdrop, the demand for real-world analysis, particularly assessing long-term outcomes, has become increasingly evident. Recognizing the need for a comprehensive assessment of long-term outcomes, retrospective analysis of cases with identical doses and identical protocols within a single institution was carried out in this study [8]. Our overarching goal is to unearth, through real-world analysis, a new idea that can steer the choice between PBT and IMRT for prostate cancer treatment for clinical physicians. Another goal is to obtain data serving a foundation for the development of Phases II and III clinical trial protocols comparing these two treatment modalities.

## MATERIALS AND METHODS

### Patients

Between 2008 and March 2018, consecutive Japanese patients with biopsy-confirmed prostate cancer from a single institution were reviewed. This retrospective study was conducted in compliance with the Declaration of Helsinki and was approved by the institutional review board of our institution (approval number: 57689). All eligible patients provided written informed consent before treatment. To protect patient privacy and data confidentiality, all personal information was anonymized, and statistical analyses were conducted using anonymized data. The study adhered to all applicable ethical guidelines and regulations.

### Eligibility criteria

Eligible patients had biopsy-confirmed adenocarcinoma of the prostate with clinical stage T1–4N0M0 and were classified into the National Comprehensive Cancer Network (NCCN)-defined (http://www.nccn.com) low-, intermediate- and high-risk groups.

### Exclusion criteria

Patients with N1 disease were excluded from the study. Patients with metastasis, other patients with concurrent invasive cancers or patients with active collagen disease were also excluded. Patients with a follow-up period of 2 years or less were also excluded from this analysis. The characteristics of the patients are listed in Table 1.

**Table 1:** Patient characteristics of the IMRT and PBT group

	All (*n* = 606)	PBT (*n* = 510)	IMRT (*n* = 96)	*P*-value
Median age Years range	67 45–84	67 45–84	70 52–83	0.001
BMI SD	24.4 3.3	24.5 3.3	25.2 3.7	0.16
Gleason score 5 6 7 8 9 10	2 (0.3%) 118 (19.5%) 299 (49.3%) 97 (16.0%) 76 (12.6%) 14 (2.3%)	2 (0.4%) 110 (21.6%) 244 (47.8%) 78 (15.3%) 62 (12.2%) 14 (2.7%)	0 8 (8.3%) 55 (57.3%) 19 (19.8%) 14 (14.6%) 0	0.004
Clinical T-score 1 2 3 4 Unclear	244 (40.3%) 260 (42.9%) 96 (15.8%) 5 (0.8%) 1(0.2%)	206 (40.4%) 225 (44.1%) 73 (14.3%) 5 (1.0%) 1(0.2%)	38 (39.6%) 35 (36.5%) 23 (23.9%) 0	0.11
NCCN risk group Low Intermediate High Very high	71 (11.7%) 270 (44.6%) 220 (36.3%) 45 (7.4%)	69 (13.5%) 228 (44.7%) 176 (34.5%) 37 (7.3%)	2 (2.0%) 42 (43.8%) 44 (45.8%) 8 (8.3%)	0.0018
PSA (ng/ml) Range	9.5 3.0–785	9.5 3.34–785	9.5 3.0–121	0.17
ADT With Without	371 (61.2%) 235 (38.8%)	302 (59.2%) 208 (40.8%)	69 (71.9%) 27 (28.1%)	0.017
Nodal risk (%) ≥40 <40	64 (10.6%) 542 (89.4%)	55 (10.8%) 455 (89.2%)	9 (9.4%) 87 (90.6%)	0.68
Heart disease With Without	90 (14.9%) 516 (85.1%)	79 (15.5%) 431 (84.5%)	11 (11.5%) 85 (88.5%)	0.29
Brain stroke With Without	34 (5.6%) 572 (94.4%)	26 (5.1%) 484 (94.9%)	8 (8.3%) 88 (91.7%)	0.23
Hypertension With Without	259 (42.7%) 347 (57.3%)	226 (44.3%) 284 (55.7%)	33 (34.4%) 63 (65.6%)	0.068
Diabetes mellitus With Without	81 (13.4%) 525 (86.6%)	70 (13.7%) 440 (86.3%)	11 (11.5%) 85 (88.5%)	0.54

BMI = body mass index, PBT = proton beam therapy, IMRT = intensity-modulated radiation therapy, NCCN = National Comprehensive Cancer Network, PSA = prostate-specific antigen

### Patient decision factors and cost coverage for PBT and IMRT

The decision to use PBT or IMRT was based on the patient’s preference. The PBT patients were those who could afford the cost of PBT, which was 2883 000 yen (approximately $24 025 when converted at a rate of 1 USD = 120 JPY), either privately or through private insurance, while IMRT was performed using public insurance. The cases in this study were from a period when PBT was not covered by Japan’s public insurance. The IMRT groups were primarily patients from the surrounding medical service areas, whereas the PBT groups were patients who came from a wide area across Japan.

### Radiation planning

#### IMRT

A total of 96 patients received IMRT delivered using standard seven coplanar beams or one–two full arcs. Treatment planning CT simulation was conducted with LightSpeed RT16 (GE Healthcare, Milwaukee, WI) set to a 2.5 mm slice thickness. In total, 10 MV photon treatments were delivered by Clinac iX linear accelerator (Varian Medical Systems, Palo Alto, CA). Each plan was generated in Eclipse (release 6.5; Varian Medical Systems, Palo Alto, CA, USA) and optimized as per our institutional constraints. The prescribed dose was 70–78 Gy to cover 50% of the target volume, with a maximum permissible dose heterogeneity within 10% planning target volume (PTV). All patients were treated with their prescribed 2.0 Gy daily fraction dose. Our treatment planning constraints for this modality were V40 < 65%, V60 < 30%, V70 < 20% and V78 < 1% for the rectum and V40 < 60% and V70 < 35% for the bladder. Dose reductions of up to 5% were permitted depending on complications and the involvement of risk organs. Patients were treated in the supine position and immobilized with a customized vacuum device. Urinary catheters, rectal balloons, gold fiducial markers and hydrogel spacers were not used before the acquisition of treatment planning. For every treatment fraction, patients were repositioned using Varian On-Board Imager based on positions of the prostate.

#### PBT

PBT treatment planning and delivery techniques were conducted for 510 patients who underwent passive-scattering proton therapy using PBT system (Mitsubishi Electric, Kobe, Japan), similar to a previous study [9]. Treatment planning was performed using CT simulation with Aquilion LB (Toshiba Medical Systems, Tokyo, Japan) set to a 2.0 mm slice thickness. The prescribed dose was 70–78 Gy (RBE), covering 95% PTV in 2-Gy (RBE) fractions. After 56 Gy (RBE), the multi-leaf collimator on the rectal side was closed, and the treatment plan was adjusted to avoid creating a high-dose zone on the anterior rectal wall. The treatment planning was optimized based on our institutional constraints: V40 < 35% and V65 < 17% for the rectum and V40 < 50% and V65 < 25% for the bladder. Dose reductions of up to 5% were permitted depending on complications and the involvement of risk organs. Other dose constraints were planned by comparing IMRT dose constraints. Patients were treated in the supine position and immobilized with a customized vacuum device. Urinary catheters, rectal balloons, gold fiducial markers and hydrogel spacers were not used before the acquisition of treatment planning CT. Two-dimensional orthogonal X-rays were used for positioning during PBT at our institution. Changes in organ volume during treatment were evaluated using ongoing verification CT scans performed with a Discovery ST Elite system (GE Medical Systems, WI, USA) immediately after PBT.

### Contouring

For low-risk patients, clinical target volume (CTV) included only the prostate. For the intermediate- and high-risk groups, CTV incorporated the base of the seminal vesicle, whereas for T3b patients, the entire seminal vesicle was included in the CTV. The CTV was expanded in three dimensions with a 0.7-cm margin to obtain the PTV, except for the prostate–rectum interface, where a 0.6-cm margin was adopted to decrease rectal involvement. The rectum, bladder, bowel and femur were contoured as the critical normal tissue structures.

### Hormonal therapy

Androgen deprivation therapy (ADT) was administered at the discretion of the treating physician. Among 539 patients receiving ADT, the median duration of ADT was 21 months (range, 1–132 months). In the PBT group, there were 213 high-risk cases, all of which (100%) received endocrine therapy. Among the 228 intermediate-risk cases, 209 cases (91.7%) received endocrine therapy, and 19 cases (8.3%) did not. For the 69 low-risk cases, 25 cases (36.2%) received endocrine therapy, while 44 cases (63.8%) did not. The duration of endocrine therapy in the PBT group for high-risk patients was a median of 26 months (range: 1–132 months), and for intermediate-risk patients, it was a median of 6 months (range: 1–71 months). In the IMRT group, there were 52 high-risk cases, with 51 cases (98.1%) receiving endocrine therapy and 1 case (1.9%) not receiving endocrine therapy. Among the 42 intermediate-risk cases, 41 cases (97.6%) received endocrine therapy and 1 case (2.4%) did not. The 2 low-risk cases (100%) did not receive endocrine therapy. The duration of endocrine therapy in the IMRT group for high-risk patients was a median of 25 months (range: 2–82 months), and for intermediate-risk patients, it was a median of 9 months (range: 1–59 months).

### Follow-up evaluation after treatment

Follow-up evaluations were conducted at 3-to 6-month intervals for 5 years following the completion of treatment and at intervals of 6–12 months thereafter. The median follow-up duration was 86 months (range, 27–171 months). Biochemical relapse-free survival (bRFS) was assessed according to the Phoenix consensus definition, which defines biochemical relapse as an absolute nadir prostate-specific antigen (PSA) level plus 2 ng/mL higher than the recorded level [7, 8]. Acute and late toxicity data were scored according to the National Cancer Institute-designated Common Terminology Criteria for Adverse Events Version 5.0.

### Statistical analyses

Categorical variables were compared between the treatment groups using the χ2 test or Fisher’s exact test, and continuous data were compared using the Mann–Whitney U test. All survival times were measured from the start of PBT or IMRT.

Kaplan–Meier curves were used to assess the distribution of 7-year bRFS for biochemical control using one failure definition. In addition, Kaplan–Meier curves were used to evaluate the 7-year actuarial disease-free survival (DFS), cause-specific survival (CSS) and overall survival (OS) rates. Univariate analysis (UA) and multivariate analysis (MA) were conducted to identify predictors of bRFS, including age, tumor stage, GS, pretreatment PSA level, treatment year, NCCN risk group, ADT use and pelvic node involvement risk estimated using the Roach formula [10]. MA was performed using the Cox regression model. Statistical analyses were performed using the JMP v16 (SAS Institute Inc, Cary, NC, USA). A *P*-value <0.05 (two-sided) was considered statistically significant in all tests.

To mitigate selection bias in the allocation of treatment groups, propensity score modeling was used to match the two groups using a logistic regression approach. Propensity score matching (PSM) was performed using a multivariate logistic regression model to predict PBT vs IMRT by incorporating covariates. Matching was conducted using 1:1 nearest-neighbor matching with a caliper of 0.2 × propensity score. The covariate sets used for propensity score modeling included age, tumor stage, GS, pretreatment PSA level, treatment year, ADT use, NCCN risk group and risk of pelvic node involvement [11, 12].

## RESULTS

### Biochemical relapse-free survival rate of analysis of all cases

The median follow-up duration for survivors was 82 months (range, 32–140 months) in the IMRT group and 87 months (range, 27–171 months) in the PBT group. At the time of analysis, PSA failure occurred in 12 of 96 patients (12.5%) in the IMRT group and 34 of 510 patients (6.7%) in the PBT group.

**Fig. 1:**
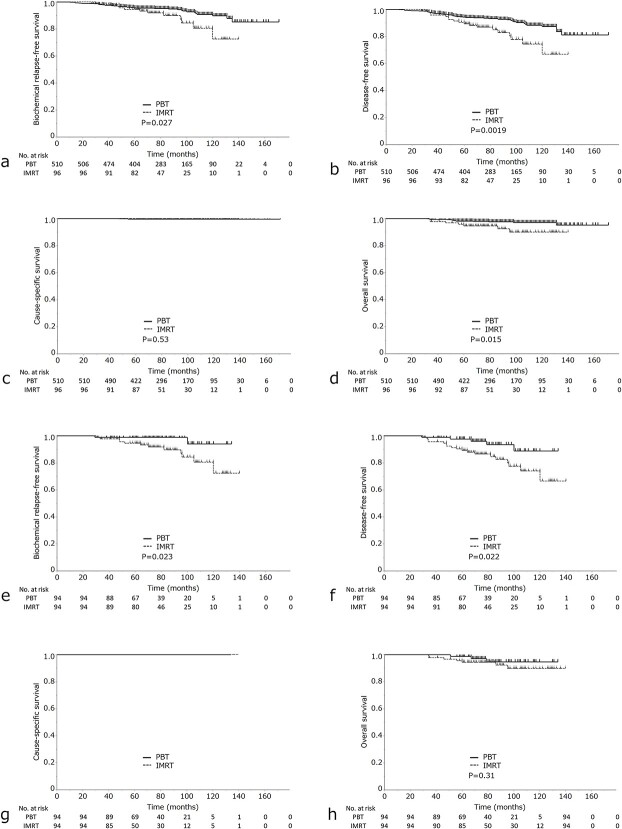
(a) bRFS, (b) DFS, (c) CSS and (d) OS) in the IMRT group vs PBT group. (e) bRFS, (f) DFS, (g) CSS and (h) OS in the IMRT and PBT group after propensity score analysis.

The PBT group had a significantly higher 7-year bRFS rate than the IMRT group [95.1% (95% CI, 92.7–96.5) for PBT vs 89.9% (95% CI, 80.8–95.0%) for IMRT; *P* = 0.027] (Fig.1a). UA showed that the significant variables were NCCN risk classification, clinical T-stage, Gleason score (GS), pretreatment PSA, nodal risk and PBT vs IMRT. Nodal risk and PBT vs IMRT remained statistically significant variables in the MA (Table 2).

**Table 2:** Univariate and multivariate analyses

	Univariate			Multivariate		
Parameter	bRFS at 7 years (%)	95% CI	*P*-value	HR	95% CI	*P*-value
Age >75 ≤75	91.2 94.6	80.4–96.3 92.2–96.3	0.78			
PS ≤1 >1	94.6 94.2	87.7–97.7 91.6–96.1	0.46			
NCCN Low-intermediate risk High risk	90.1 88.6	86.4–93.8 81.3–93.4	0.015	1.05	0.46–2.40	0.90
T stage T1–T2 T3–T4	95.8 86.6	93.4–97.4 78.3–92.0	0.010	1.24	0.56–2.71	0.59
Gleason score ≤8 >8	96.0 84.1	93.7–97.5 74.3–90.6	0.0002	1.52	0.60–3.87	0.35
PSA (ng/ml) ≤50 >50	95.1 77.9	92.9–96.7 58.5–89.8	0.0043	1.24	0.39–3.98	0.72
Radiation therapy PBT IMRT	95.1 89.9	92.7–96.7 80.8–95.0	0.027	2.20	1.13–4.33	0.021
Durations of ADT (month) ≥24 <24	92.7 94.9	87.2–95.9 92.1–96.7	0.45			
Nodal risk (%) ≥40 <40	96.1 78.3	94.0–97.6 96.0–87.0	<0.0001	3.87	1.33–11.2	0.013
Private insurance coverage for PBT With coverage Without coverage	94.9 94.0	90.1–97.4 91.0–96.0	0.94			
Heart disease With Without	93.6 94.3	85.5–73.3 91.3–96.2	0.27			
Brain stroke With Without	92.9 94.4	75.6–98.2 92.0–96.1	0.66			
Hypertension With Without	96.0 93.2	92.4–97.9 89.9–95.5	0.54			
Diabetes mellitus With Without	95.5 94.1	86.9–98.5 91.5–95.9	0.69			
Smoking With Without	97.0 92.7	93.6–98.7 89.4–95.0	0.10			
Alcoholic history With Without	95.2 93.6	91.7–97.2 90.0–96.0	0.88			

bRFS = biochemical relapse-free survival, NCCN = National Comprehensive Cancer Network, PSA = prostate-specific antigen, PBT = proton beam therapy, IMRT = intensity-modulated radiation therapy, ADT = androgen deprivation therapy

### DFS, CSS and OS of analysis of all cases

The PBT group showed a significantly higher 7-year DFS rate than the IMRT group [93.1% (95% CI, 90.4–95.1%) for PBT vs 85.0% (95% CI, 75.5–91.2%) for IMRT; *P* = 0.0019] (Fig. 1b). The 7-year CSS rates were 100 and 99.5% in the IMRT and PBT groups, respectively (Fig. 1c), with no statistically significant difference. The IMRT group showed a significantly lower 7-year OS rate than the PBT group [97.5% (95% CI, 95.5–98.6%) for PBT vs 94.6% (95% CI, 87.6–97.8%) for IMRT; *P* = 0.015] (Fig. 1d). In the PBT group, there were nine cases of non-cancer-related deaths. Of these, one case was due to lung cancer, one case was due to pneumonia, and the remaining seven cases were due to old age or other unclear causes. In the IMRT group, there were six cases of non-cancer-related deaths. Of these, four cases were due to lung cancer, one case was due to myocardial infarction, and the cause of death was unknown for one case.

### Toxicity

Eight patients (8.3%) were taking oral anticoagulants or antiplatelet medications for IMRT and 53 patients (10.4%) were taking them for PBT. In both groups, there were no cases of acute GI and GU toxicity of grade 3 or higher. In the IMRT group, rectal bleeding (late grade 2 GI toxicity) occurred in seven patients (7.3%). Rectal bleeding (late grade 2 or 3 GI toxicity) occurred in 13 patients (2.6%) in the PBT group. Hyperbaric oxygenation or argon plasma coagulation (APC) was not required in any patient in the IMRT group. However, in the PBT group, APC was used in four cases to address rectal bleeding, and hyperbaric oxygenation was used in two cases.

### Matched analysis

We performed using logistic regression analysis. After matching, 94 patients were assigned to the PBT group and 94 patients were assigned to the IMRT group. Patient characteristics are shown in Table 3. After PSM, the mean propensity score was 0.80 (± 0.075) in the PBT group and 0.80 (± 0.074) in the IMRT group. Eight patients (8.5%) were taking oral anticoagulants or antiplatelet medications for IMRT and 11 patients (11.7%) were taking them for PBT. The PBT group showed a significantly higher 7-year rate of bRFS than the IMRT group [98.9% (95% CI, 92.7–99.8%) for PBT vs 89.7% (95% CI, 80.3–94.9%) for IMRT; *P* = 0.023] (Fig. 1e) and a significantly higher 7-year rate of DFS than the IMRT group [93.4% (95% CI, 83.2–97.7%) for PBT vs 84.6% (95% CI, 75.0–91.0%) for IMRT; *P* = 0.022] (Fig. 1f). However, no statistically significant difference was found between the two groups in OS [94.6% (95% CI, 84.1–98.3%) for PBT vs 94.5% (95% CI, 87.4–97.7%) for IMRT; *P* = 0.31] or CSS (*P* = 0.8) (Fig. 1g, h).

**Table 3:** Patient characteristics after PSM

	All (*n* = 188)	PBT (*n* = 94)	IMRT (*n* = 94)	*P*-value
Median age Years range	69 52–83	68 55–80	70 52–83	0.85
BMI SD	24.7 3.3	24.4 3.2	24.7 3.6	0.16
Gleason score 5 6 7 8 9 10	0 19 (10.11%) 107 (56.9%) 34 (18.1%) 28 (14.9%) 0	0 11 (11.7%) 54 (57.5%) 15 (15.9%) 14 (14.9%) 0	0 8 (8.5%) 53 (56.4%) 19 (20.2%) 14 (14.9%) 0	0.81
Clinical T-score 1 2 3 4 Unclear	73 (38.8%) 77 (41.0%) 38 (20.2%) 0 0	35 (37.2%) 42 (44.7%) 17 (18.1%) 0 0	38 (40.4%) 35 (37.2%) 21 (22.4%) 0 0	0.55
NCCN risk group Low Intermediate High Very high	4 (2.1%) 87 (46.3%) 85 (45.2%) 12 (6.4%)	2 (2.1%) 45 (47.9%) 42 (44.7%) 5 (5.3%)	2 (2.1%) 42 (44.7%) 43 (45.8%) 7 (7.4%)	0.94
PSA (ng/ml) Range	8.9 3.0–146	8.6 3.34–146	9.0 3.0–121	0.34
ADT With Without	130 (69.2%) 58 (30.8%)	63 (67.0%) 31 (32.9%)	67 (71.3%) 27 (28.7%)	0.52
Nodal risk (%) ≥40 <40	14 (7.5%) 174 (92.5%)	5 (5.3%) 89 (94.7%)	9 (9.6%) 85 (90.4%)	0.26
Private insurance coverage for PBT With coverage Without coverage	66 (35.1%) 122 (64.9%)	38 (40.4%) 56 (59.6%)	28 (29.8%) 66 (70.2%)	0.12
Heart disease With Without	26 (13.8%) 162 (86.2%)	15 (16.0%) 79 (84.0%)	11 (11.7%) 83 (88.3%)	0.39
Brain stroke With Without	11 (5.9%) 177 (94.1%)	3 (3.2%) 91 (96.8%)	8 (8.5%) 86 (91.5%)	0.11
Hypertension With Without	73 (38.8%) 115 (61.2%)	40 (42.6%) 54 (57.4%)	33 (35.1%) 61 (64.9%)	0.29
Diabetes mellitus With Without	18 (9.6%) 170 (90.4%)	7 (7.5%) 87 (92.5%)	11 (11.7%) 83 (88.3%)	0.31

BMI = body mass index, PBT = proton beam therapy, IMRT = intensity-modulated radiation therapy, NCCN = National Comprehensive Cancer Network, PSA = prostate-specific antigen, ADT = androgen deprivation therapy

The cumulative incidence of grade 2 or higher late GI and GU in the IMRT group was 2.6% (95%CI, 0.4–16.1%) and 1.5 (95% CI, 0.2–10.1%), respectively, at 7 years, whereas that in the PBT group was 5.1% (95% CI, 1.9–13.3%) and 3.9% (95% CI, 0.9–16.1%), respectively, at 7 years. There was no significant difference in the cumulative incidence of grade 2 or higher late GI or GU between the PBT and IMRT groups (*P* = 0.06 and *P* = 0.20, respectively). A comprehensive dose–volume histogram comparison between 93 PBT and 94 IMRT dates revealed distinct differences in dose distribution. Although matched cases were used to evaluate toxicity, one PBT case lacked historical data, resulting in the analysis of 93 cases (Table 4).

**Table 4:** Dose parameters of PTV, CTV, rectum and bladder for PBT (*n* = 93) and IMRT (*n* = 94)

	PBT (*n* = 93)	IMRT (*n* = 94)	*P*-value
PTV (cc)	80.1 ± 21.4	83.7 ± 24.2	0.39
Mean dose [Gy (RBE) or Gy]	74.2 ± 2.4	75.1 ± 2.5	0.0013
Minimal dose [Gy (RBE) or Gy]	51.4 ± 10.1	55.7 ± 6.4	0.0005
Max dose [Gy (RBE) or Gy]	77.4 ± 2.7	80.1 ± 3.2	< 0.0001
D2cc [Gy (RBE) or Gy]	77.0 ± 2.6	78.4 ± 2.8	0.0016
D98% [Gy (RBE) or Gy]	63.2 ± 6.3	66.3 ± 3.4	< 0.0001
D50 [Gy (RBE) or Gy]	75.6 ± 2.4	75.8 ± 2.6	0.0052
D2% [Gy (RBE) or Gy]	77.0 ± 2.6	78.5 ± 2.8	0.0015
CTV (cc)	27.8 ± 10.3	28.6 ± 11.9	0.89
Mean dose [Gy (RBE) or Gy]	76.1 ± 2.2	76.6 ± 2.4	0.0028
Minimal dose [Gy (RBE) or Gy]	72.8 ± 3.8	73.0 ± 0.87	0.04
Max dose [Gy (RBE) or Gy]	77.3 ± 2.6	79.2 ± 2.7	< 0.0001
D2cc [Gy (RBE) or Gy]	76.7 ± 2.4	77.7 ± 2.5	0.0013
D98% [Gy (RBE) or Gy]	74.7 ± 2.7	74.4 ± 2.5	0.47
D50 [Gy (RBE) or Gy]	76.0 ± 2.4	76.5 ± 2.4	0.0028
D2% [Gy (RBE) or Gy]	77.3 ± 2.4	78.5 ± 2.5	0.0008
Rectum (cc)	43.7 ± 12.7	43.5 ± 18.8	0.29
V10 (%)	47.5 ± 10.2	85.3 ± 18.0	< 0.0001
V20 (%)	36.7 ± 8.6	63.7 ± 21.2	< 0.0001
V30 (%)	29.6 ± 6.7	39.8 ± 15.3	< 0.0001
V40 (%)	23.1 ± 5.9	25.5 ± 9.7	0.079
V50 (%)	17.1 ± 5.2	17.5 ± 6.8	0.78
V60 (%)	11.1 ± 4.4	11.2 ± 5.0	0.99
V70 (%)	4.4 ± 3.2	4.5 ± 3.0	0.68
Mean dose [Gy (RBE) or Gy]	20.4 ± 4.4	29.9 ± 6.5	< 0.0001
Bladder (cc)	151.5 ± 100.0	158.7 ± 91.4	0.25
V10 (%)	49.6 ± 21.1	62.5 ± 24.2	0.0002
V20 (%)	41.3 ± 18.9	49.6 ± 22.1	0.01
V30 (%)	35.6 ± 16.5	38.4 ± 18.5	0.34
V40 (%)	29.7 ± 14.5	28.7 ± 14.2	0.67
V50 (%)	24.0 ± 12.4	20.5 ± 10.4	0.082
V60 (%)	18.1 ± 10.1	14.4 ± 7.6	0.024
V70 (%)	10.5 ± 7.3	8.5 ± 4.8	0.16
Mean dose [Gy (RBE) or Gy]	24.2 ± 10.9	27.1 ± 10.0	0.063

CTV = clinical target volume, D2cc = dose to the hottest 2 cm, D2% = dose received by 2% of volume, D50 = dose received by 50% of volume; median dose; D98% = dose received by 98% of volume, IMRT = intensity-modulated radiation therapy, PBT = proton beam therapy, PTV = planning target volume. Data are presented as mean ± standard deviation, RBE = relative biological effectiveness.

### Subgroup analysis by NCCN risk of analysis of all cases

**Fig. 2:**
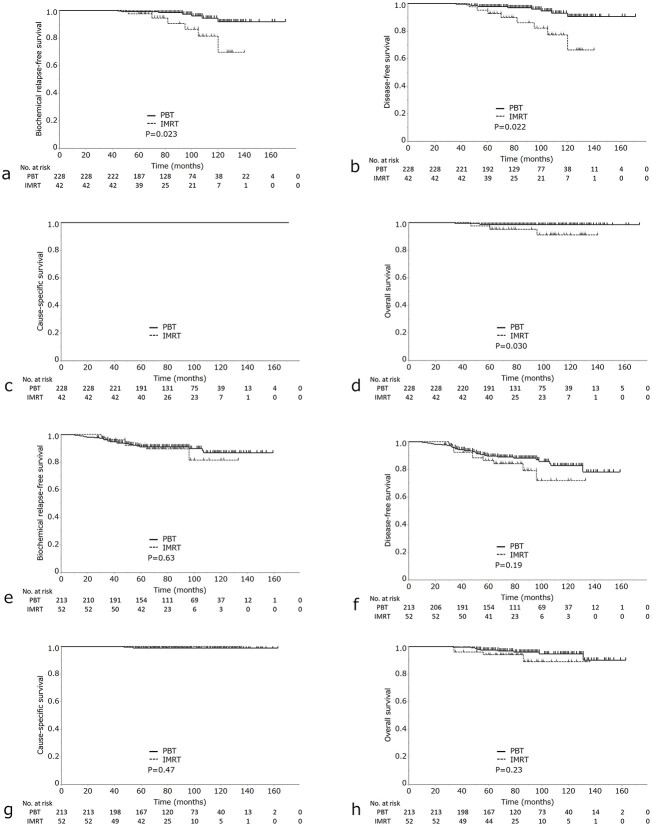
(a) bRFS, (b) DFS, (c) CSS and (d) OS in high-risk prostate cancer in the IMRT group vs the PBT group. (e) bRFS, (f) DFS, (g) CSS and (h) OS in intermediate-risk prostate cancer in the IMR vs the PBT groups.

A subgroup analysis was performed to investigate the bRFS, DFS, CSS and OS of the intermediate- and high-risk groups (Fig. 2a–h). Intermediate-risk group patients in the IMRT group showed a significantly lower 7-year bRFS rate than that in intermediate-risk patients in the PBT group [98.3% (95% CI, 94.8–99.5%) for PBT vs 90.5% (95%CI, 73.9–97.0%) for IMRT; *P* = 0.007] (Fig.2a). The IMRT group also had significantly lower 7-year OS rates [98.6% (95%CI, 95.8–99.5%) for PBT vs 95.1% (95% CI, 82.4–98.8%) for IMRT; *P* = 0.032] (Fig. 2d). However, there was no significant difference between high-risk patients in the IMRT and PBT groups in the 7-year bRFS or OS (Fig. 2e, h).

## DISCUSSION

Patients who received PBT showed a significantly higher 7-year bRFS and a significantly higher DFS rate, with and without PSM. Considering the numerous reports suggesting the limited treatment efficacy of PBT, including its cost effectiveness [4, 6, 13, 14], the findings of this study are important. The better outcomes may be partially explained by the dosimetric advantages of PBT over IMRT [8]. First, the physical advantage of the Bragg peak is that it allows the photon dose to be focused within the target, minimizing the dose to adjacent organs [8, 15]. Furthermore, PBT and IMRT were performed at the same facility using the same protocol. At the same prescription dose, the high biological efficacy of PBT, characterized by RBE >1.1 [16], may have contributed to the enhanced local control of PBT and may have led to a greater biological efficacy of PBT than that of IMRT. Previous reports have also highlighted the potential effectiveness of PBT in prostate cancer treatment [15, 17], attributed to its high precision in radiation delivery and lower radiation transmission to surrounding tissues compared to IMRT [18, 19].

IMRT is a standard treatment for localized prostate cancer with a 5-year bRFS of approximately 90% [13, 17–19]. In this study, there was a substantial representation of patients in the intermediate- and high-risk groups, mirroring a patient distribution similar to that observed in real-world clinical practice. The results are comparable to or better than those of previous IMRT studies [20–23]. According to previous studies, the prescription dose range was 70.2–78 Gy/Gy (RBE), and the 5-year bRFS rate ranged from 80 to 90.5%. PBT operates within a prescription dose range of 70 to 80 Gy/Gy (RBE) and boasts 5-year bRFS rates ranging from 82 to 99% [24–32]. Supplementary Table S1 shows the treatment outcomes of 3DCRT, IMRT and PBT in previous studies using irradiation in conventional fractionation, which are comparable to those in this study.

Subgroup analysis according to NCCN risk revealed the advantages of PBT in the intermediate-risk group in this study. Patients with intermediate-risk prostate cancer have fewer lymph node and distant metastases than those with high-risk prostate cancer, which may have led to the pronounced biological effects of PBT local prostate cancer treatment [8]. A multicenter trial of PBT for intermediate-risk prostate cancer was initiated in Japan and relevant reports have been published [33]. The bRFS rates in the patients with high-risk prostate cancer were not significantly different between the PBT and IMRT groups. One possible reason for this may be an inadequate local dose. There have been reports of IMRT outcomes with doses exceeding 80 Gy [34–36]. In addition, very high-risk cases increase the frequency of lymph node and bone metastases; other effects besides local control should also be considered [16].

Supplementary Table S2 summarizes the previous records of the side effects of PBT and IMRT. The frequencies of late GI and GU toxicities in our study were similar to those reported previously [25, 26, 28–30, 37–40]. Notably, no significant difference was observed in the frequency of late GI toxicity between the PBT and IMRT groups. In fact, for the rectum, although the frequency was lower with PBT, it resulted in stronger side effects, with some cases requiring APC or high-pressure oxygen, as in previous studies [41]. The fact that more patients in the PBT group were on anticoagulants and antiplatelet medications may also have influenced the results.

The present study has some limitations. Two-dimensional orthogonal X-rays were used for positioning during PBT at our institution. Currently, in many PBT facilities, image guidance is moving toward online volumetric cone beam CT (CBCT) [31]. The dose concentration of PBT may not have been as favorable as that of IMRT using CBCT [8, 42, 43]. There was no statistically significant difference in late GU toxicity between patients who underwent PBT and those who underwent IMRT. In addition to the effects of anticoagulants and antiplatelet agents, PBT showed notably higher values than IMRT for V60 of the bladder in the present study, which might also have affected the frequency of late GU toxicity of PBT [44]. With the implementation of hypofractionated irradiation, there is ample potential to address these issues [45]. Because this was a retrospective study, there was a possibility of information bias and limitations due to reliance on existing medical records and databases, which might have contained missing or incomplete information. Imaging studies to assess distant metastasis were not uniformly performed in all cases, making it impossible to calculate distant metastasis-free survival. To mitigate these selection biases in the allocation between IMRT and PBT, PSM was used to match the two groups using a logistic regression approach. However, PSM also reduced the number of patients in the PBT group. A smaller sample size could diminish the statistical power of the study, making it difficult to detect minor or significant associations. There are also seven variables that our list, where some have co-dependence, e.g. NCCN risk group, PSA, Gleason and clinical stage, and the total number of events (PSA failure) are likely to low to perform a robust multivariable regression in this present study. There remain considerable concerns when comparing outcomes with other modalities such as IMRT in a non-randomized retrospective review, even after PSM [46, 47]. Furthermore, due to limitations in the statistical analysis software available at our facility (JMP), we were unable to use Fine and Gray’s regression model to account for competing risks when recalculating bRFS and DFS rates using the cumulative incidence function [48].

Although it is difficult to eliminate bias in this study, it is important to consider these factors when interpreting the results. Furthermore, a limitation of this study is that specific adjunctive devices or techniques (e.g. gold fiducial markers or hydrogel spacers) that are currently used were not used, which might have caused differences compared to current treatment methods. These adjunctive devices or techniques can contribute to treatment precision and the management of side effects. Therefore, further research incorporating these elements is required. It is important to note that the results represent treatment outcomes from a generation ago, including passive-scattering proton therapy, which is not reflective of current mainstream practice. The results might not be perceived as clinically important, as mainstream proton therapy is now delivered using pencil beam scanning [49, 50].

This study has also several limitations related to patient background and economic status. First, many patients in the IMRT group came from the surrounding medical regions, particularly from areas with survival rates lower than the national average, which may have influenced the survival rates in this study [51]. Additionally, IMRT patients were more likely to have only public insurance, which could affect their overall health outcomes and access to supportive care. Conversely, PBT patients came from a broader geographic area in Japan and were less affected by specific regional characteristics. They also tended to have private insurance and higher financial solvency, which may have provided them with better access to healthcare resources and support. Previous studies have shown that differences in financial capacity can significantly impact treatment outcomes [52].

This study showed that the treatment outcomes of PBT for prostate cancer potentially surpassed those of IMRT, particularly concerning bRFS and DFS. This emphasizes the potential value of PBT as a treatment option for patients. Attention is warranted for adverse events of PBT, particularly those affecting the bladder.

## Supplementary Material

Table_S1_ITA_YOUKWJ-15_clean_rrae065

Table_S2_ITA_YOUKWJ-15_rrae065

## AUTHOR RESPONSIBLE FOR THE STATISTICAL ANALYSES

Yojiro Ishikawa.

## PRESENTATION AT A CONFERENCE

Presented at the 36th Annual Meeting of the Japanese Society for Radiation Oncology in 2023.

## DATA STATEMENT

The datasets analyzed in the current study are available from the corresponding author on reasonable request.
