# Supplementary material for: Real-world comparative outcomes and toxicities after definitive radiotherapy using proton beam therapy versus intensity-modulated radiation therapy for prostate cancer: a retrospective, single-institutional analysis
Source: J Radiat Res. 2025 Jan 15;66(1):39–51. doi: 10.1093/jrr/rrae065 (PMC11753839; doi:10.1093/jrr/rrae065)
Supplement: Table_S1_ITA_YOUKWJ-15_clean_rrae065 [file table_s1_ita_youkwj-15_clean_rrae065.docx]

|  | Year | Irradiation techniques | Number of patients | Prescription dose [Gy/Gy (RBE)] | LR | IR | HR |
| --- | --- | --- | --- | --- | --- | --- | --- |
|  |  |  |  |  | 5-year bRFS rate | | |
| D’Amico[25] | 2002 | 3DCRT | 381 | 66–70 | 80 | 65–75 | 40 |
| Dearnaley [26] | 2007 | 3DCRT | 422 | 74 | 85 | 79 | 57 |
| Vora [27] | 2007 | IMRT | 145 | 70.2–77.4 | 80 | 65–70 | 40 |
| Shula [28] | 2000 | PBT | 911 | 74–75 | 82 | 82 | 82 |
| Takagi [29] | 2017 | PBT | 1,375 | 74 | 99 | 91 | 86 |
| Bryant [30] | 2016 | PBT | 1,327 | 72–78 | 99 | 94 | 74 |
| Iwata [31] | 2018 | PBT | 1,291 | 70–80 | 97 | 91 | 83 |
| Arimura [32] | 2018 | PBT | 218 | 70 | NR | 97 | 83 |
| Ho [33] | 2018 | PBT | 252 | 76–82  70–72.5 | 97.8 | 97.89  7.8 | 97.8 |
| Present study for IMRT group | 2023 | IMRT | 94 | 70–78 | 100* | 90.5 | 89.6 |
| Present study for PBT group | 2023 | PBT | 510 | 70–78 | 96.9 | 98.3 | 89.7 |

Table S1. Literature review of biochemical relapse-free survival rate

LR, low-risk; IR, intermediate-risk; HR, high-risk; bRFS, biochemical relapse-free survival; 3DCRT, three conformal radiation therapy; IMRT, intensity modulated radiation therapy; PBT, proton beam therapy; GI, gastrointestinal; GU, genitourinary; RBE, relative biological effectiveness

*There were only two low-risk patients in the IMRT group in this study.
