# Supplementary material for: Real-world comparative outcomes and toxicities after definitive radiotherapy using proton beam therapy versus intensity-modulated radiation therapy for prostate cancer: a retrospective, single-institutional analysis
Source: J Radiat Res. 2025 Jan 15;66(1):39–51. doi: 10.1093/jrr/rrae065 (PMC11753839; doi:10.1093/jrr/rrae065)
Supplement: Table_S2_ITA_YOUKWJ-15_rrae065 [file table_s2_ita_youkwj-15_rrae065.docx]

Table S2. Literature review of toxicity

|  | Year | Irradiation techniques | Number of patients | Prescription dose [Gy/Gy (RBE)] | Late GI | | | Late GU | | |
| --- | --- | --- | --- | --- | --- | --- | --- | --- | --- | --- |
|  |  |  |  |  | grade1 | grade2 | grade3 | grade1 | grade2 | grade3 |
| Huang [38] | 2002 | 3DCRT | 163 | 74–78 |  | 21 | 6 |  |  |  |
| Dearnaley [26] | 2007 | 3DCRT | 843 | 74 | 45 | 14 | 4 | 14 | 6 | 2 |
| Fonteyne [39] | 2007 | IMRT | 575 | 74–80 |  | 13 |  |  |  |  |
| Vora [27] | 2007 | IMRT | 145 | 75.6 | 20 | 23 | 1 | 27 | 23 | 6 |
| Slater [40] | 1998 | PBT | 643 | 74–75 | - | 21 | 0 |  | 5.2 | 0.3 |
| Nihei K [41] | 2010 | PBT | 151 | 74 | 14 | 2.0 | - |  | 4.1 |  |
| Bryant [30] | 2016 | PBT | 1,327 | 72–78 |  | 0.6 |  |  | 2.9 |  |
| Takagi [42] | 2017 | PBT | 1,375 | 74 |  | 3.9 |  |  | 2.0 |  |
| Iwata [31] | 2018 | PBT | 1,291 | 70–80 | 4.1 | 0.5 |  | 4.0 | 0.3 |  |
| Present study for IMRT group | 2023 | IMRT | 96 | 70–78 |  | 8.0 |  |  | 1.5 |  |
| Present study for PBT group | 2023 | Proton | 606 | 70–78 |  | 5.7 | - |  | 4.3 |  |

3DCRT, three conformal radiation therapy; IMRT, intensity modulated radiation therapy; PBT,

proton beam therapy; GI, gastrointestinal; GU, genitourinary; RBE, relative biological effectiveness
